# Metastatic Occult Primary Lobular Breast Cancer: A Case Report

**DOI:** 10.7759/cureus.58586

**Published:** 2024-04-19

**Authors:** Athanasios Pouptsis, Julia Cano Gimeno, Carmen Martinez Rubio, Marta Bañuls Marrades, Patricia Olivan Sasot

**Affiliations:** 1 Department of Medical Oncology, Hospital Universitario de la Ribera, Valencia, ESP; 2 Department of Radiology, Hospital Universitario de la Ribera, Valencia, ESP; 3 Department of Gastroenterology, Hospital Universitario de la Ribera, Valencia, ESP; 4 Department of Nuclear Medicine, Hospital Universitario de la Ribera, Valencia, ESP

**Keywords:** lobular breast cancer, cdk 4/6 inhibitors, occult, metastatic, breast cancer, lobular

## Abstract

Breast cancer is the most common malignancy diagnosed in women. Invasive lobular breast cancer (ILC) is the second most common histologic subtype after invasive ductal carcinoma. Metastatic occult primary breast cancer, although rare, is a well-known clinical entity that usually presents with axillary lymphadenopathy without a detectable breast tumour. A perimenopausal woman in her 50s presented with abdominal pain, fatigue, and weight loss. Imaging showed peritoneal carcinomatosis with ascites, ovarian masses, and a lesion in the ascending colon. Gastric and colon biopsies showed infiltration from lobular breast cancer. Diagnostic workup, including mammography, breast ultrasound, and breast MRI, showed no evidence of breast pathology or axillary lymphadenopathy. First-line treatment with goserelin, letrozole, and palbociclib commenced with clinical improvement and radiological response. This case illustrates the challenges faced by clinicians in the diagnosis and treatment of lobular breast cancer without an identifiable primary lesion or axillary lymphadenopathy.

## Introduction

Invasive lobular breast cancer (ILC) is the second most common histologic subtype of invasive breast cancer after ductal carcinoma, as it constitutes 5%-15% of cases [1]. The incidence of ILC has increased during the last two decades, potentially due to the increased use of screening mammograms and breast MRIs [2]. However, the clinical and radiological diagnosis of ILC remains a challenge for clinicians due to the absence of typical clinical signs such as a palpable breast nodule or palpable lymphadenopathy and the decreased sensitivity of detection with mammograms, ultrasound, and MRI compared with other breast cancer subtypes [3].

Risk factors for ILC include prolonged exposure to hormones containing progesterone, advanced age [1], menarche before 12 years of age, menopause after 55 years of age, and childbearing at advanced age [4].

The majority of ILC cases are positive for oestrogen receptor (ER) and progesterone receptor (PgR) expression and negative for the overexpression of HER2, and have a low Ki-67 proliferative index [5]. The distinctive characteristic of ILC is the loss of expression of E-cadherin through different mechanisms [6]. The loss of E-cadherin expression leads to a loss of intercellular adhesion, and changes in cell morphology, and promotes invasion and metastasis [7]. This lack of cell cohesion yields the characteristic histologic features of ILC: sparse single tumour cells or linear strands of cells [8]. 

Invasive lobular breast cancer has a distinct pattern of metastatic disease, with an increased incidence of gastric (2.8%), peritoneal (14.6%), ovarian (4.5%), and leptomeningeal metastases (4%) [9]. Notably, peritoneal carcinomatosis is significantly more common among patients with ILC compared to those with invasive ductal cancer (IDC), posing a clinical challenge [2]. 

Metastatic occult primary breast cancer is a rare but well-known entity [10], described in the literature, with the most common sites of metastatic spread being the axillary lymph nodes and bones. Cases of metastatic occult primary lobular breast cancer with GI tract and axillary lymph node spread have been previously published [11-13].

Here we present the case of a perimenopausal woman, with de novo metastatic occult primary lobular breast cancer without axillary lymphadenopathy. 

## Case presentation

A 51-year-old woman presented to the accident and emergency department of our centre with chronic abdominal pain, unintentional weight loss of 20%, and worsening fatigue during a nine-month period. In the three months before her presentation, she developed progressive dysphagia to solids, early satiety, and nausea that did not improve when treated with metoclopramide. Four weeks before presentation, the patient developed changes in bowel habits with alternating diarrhoea and constipation. The patient denied any shortness of breath, cough, haemoptysis, haematuria, rectal bleeding, or fever. No recent travel was reported. 

Her previous medical history was insignificant, with only the occasional use of omeprazole. She reported an allergy to sulphonamides without any further information. She reported living with her husband and being the mother of two sons. Her age of menarche was 10 years, and her last menstrual cycle was three months before presentation. The last mammogram was done 18 months before presentation with no abnormal findings. No history of breast biopsies was reported. Her family’s oncological history included her sister’s endometrial cancer diagnosis at 40 years of age, her paternal aunt’s breast cancer diagnosis at 80 years of age, and her maternal aunt’s breast cancer diagnosis at 55 years of age.

During clinical examination, the patient was comfortable at rest, and a performance status of one was recorded. The patient’s body mass index was 19 kg/m^2^, with evidence of sarcopenia. She was afebrile with a blood pressure of 110/75 mmHg, a heart rate of 81 beats per minute, an oxygen saturation of 98% when breathing room air, and a body temperature of 36.5°C. Respiratory, cardiovascular, and brief neurological examinations were insignificant. The abdomen was soft, with tenderness on deep palpation in the umbilical region and right iliac fossa without guarding. A hard, painless mass was palpated in the right iliac fossa. Intestinal sounds were present and normal. No organomegaly was detected. No pedal oedema or evidence of deep vein thrombosis was found.

Blood testing revealed normochromic, normocytic anaemia. Thyroid-stimulating hormone was 5.8 mU/mL. Tumour markers were also requested. Cancer antigen 125 levels were 71 U/mL, and cancer antigen 15-3 levels were 114 U/mL. A faecal test for occult intestinal bleeding was positive (Table 1). The patient was subsequently admitted to the hospital for further investigation.

**Table 1:** Laboratory data

Variable	On initial evaluation	Reference range, adults
Haemoglobin (g/dL)	10.3	11.5 – 16.5
Haematocrit (%)	30.1	35-46
Mean corpuscular volume (fl)	95.1	80-96
Mean corpuscular haemoglobin (pg)	32.1	26-34
Mean corpuscular haemoglobin concentration (g/dL)	34.2	31-37
White blood cells x10e9/L	4.3	4.2-11.5
Neutrophils x10e9/L	2.6	1.5-7
Lymphocytes x10e9/L	1.2	0-1.9
Monocytes x10e9/L	0.1	0.07
Platelet count x10e9/L	227	120-450
Iron levels (mcg/dL)	121	50-170
Total iron-binding capacity (mcg/dL)	409	250-425
Ferritin (ng/dL)	52	10-291
Sodium (mmol/L)	142	136-145
Potassium (mmol/L)	4.1	3.5-5.1
Calcium (mg/dL)	9.3	8.7-10.4
Phosphate (mg/dL)	3.4	2.4-5.1
Creatinine (mg/dL)	0.82	0.55-1.02
Glucose (mg/dL)	84	74-106
Alanine aminotransferase (U/L)	64	46-116
Aspartate aminotransferase (U/L)	30	0-34
Total bilirubin (mg/dL)	0.47	0.3-1.2
Direct bilirubin (mg/dL)	0.1	0-0.3
Alkaline phosphatase (U/L)	64	46-116
C-reactive protein (mg/dl)	<0.5	0-5
Thyroid-stimulating hormone (mcU/ml)	5.857	0.55-4.78
Free thyroxine (T4) (ng/dL)	1.2	0.9-1.7
Cancer antigen 125 (U/mL)	71	0-30
Cancer antigen 15-3 (U/mL)	114	0-32
Carcinoembryonic antigen (ng/mL)	1.2	0-2.6
Alpha-fetoprotein (ng/dL)	4	0-8
Faecal occult blood test	Positive	Negative

**Figure 1:**
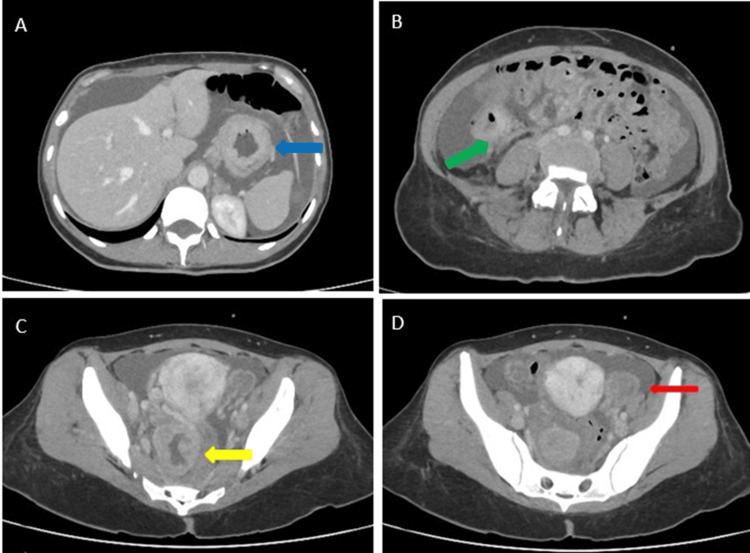
Computer tomography of the chest, abdomen, and pelvis (axial image) A regular concentric thickening of the stomach walls is observed (blue arrow). Diffuse ascites is visualised (panel A). A regular concentric thickening of the walls of the ascending colon is observed (green arrow), as well as the presence of soft tissue surrounding the vascular bundle of the mesocecum and thickening of the right posterior peritoneum, consistent with carcinomatosis (panel B). Two pelvic lesions are identified, arising from both ovaries, with one on the right measuring 5 centimetres in diameter (panel C, yellow arrow) and another on the left measuring 3 centimetres (panel D, red arrow). Both lesions exhibit solid cystic features suggestive of metastatic implants.

Computerised tomography scans of the chest, abdomen, and pelvis showed significant ascites, bilateral ovarian masses of 5 cm, wall thickening of the ascending colon with mesenterial and peritoneal infiltration, and bilateral grade 2 hydronephrosis (Figure 1). Further investigations were requested to identify the primary lesion.

**Figure 2:**
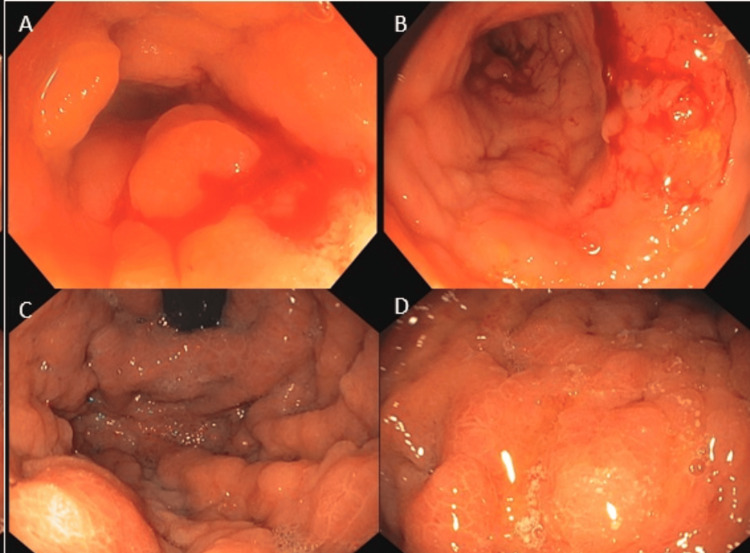
Colonoscopy and gastroscopy images At the level of the hepatic angle, 80 centimetres from the anal margin, circumferential involvement of the mucosa is observed, affecting the entire diameter and trapping the colonoscope. Oedematous mucosa with loss of the usual pattern is also noted (panels A and B). A striking lack of distensibility at the level of the gastric body, with congested and heterogeneous-looking mucosa, is likely infiltrative (panels C and D).

A colonoscopy was then performed. At the hepatic angle, a mucosal lesion was revealed that could not be passed by the colonoscope (Figures 2A -2B). The lesion was biopsied. An esophagogastroduodenoscopy showed a lack of distensibility of the gastric body with a heterogeneous appearance of the mucosa (Figures 2C-2D). Several biopsies were taken from the gastric body.

**Figure 3:**
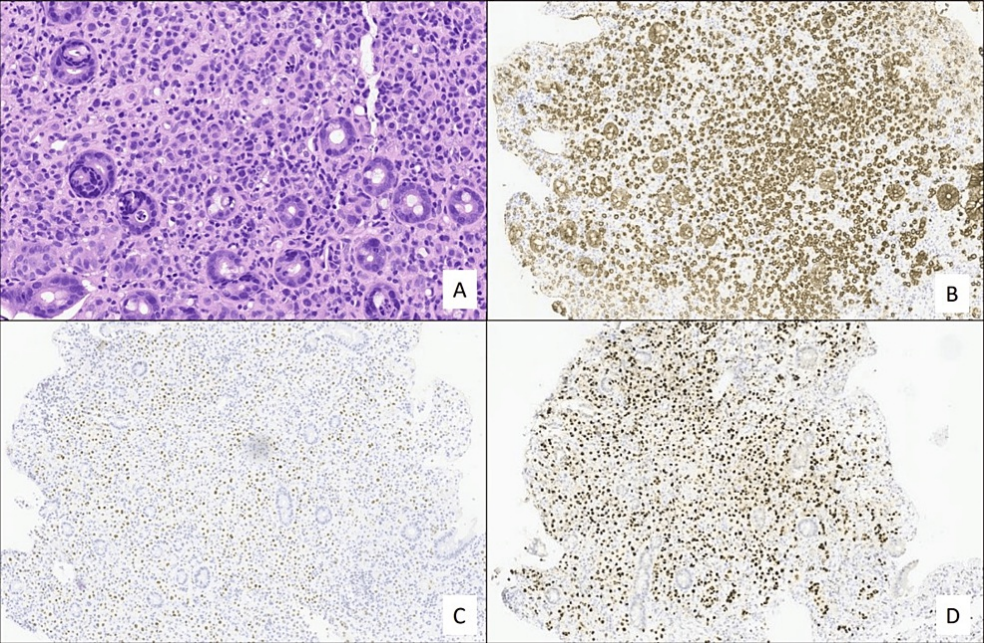
Colon biopsies A) haematoxylin-eosin; B) cytokeratin Ae1ae3; C) estrogen receptor; D) GATA 3

Colon biopsies showed infiltration from adenocarcinoma with a discohesive pattern (Figure 3). Immunochemistry was positive for CK7, mammaglobin, GATA, GCDF15, ER, and PgR and negative for CK20, CDX2, PAX8, and WT1. There was no overexpression of HER2.

**Figure 4:**
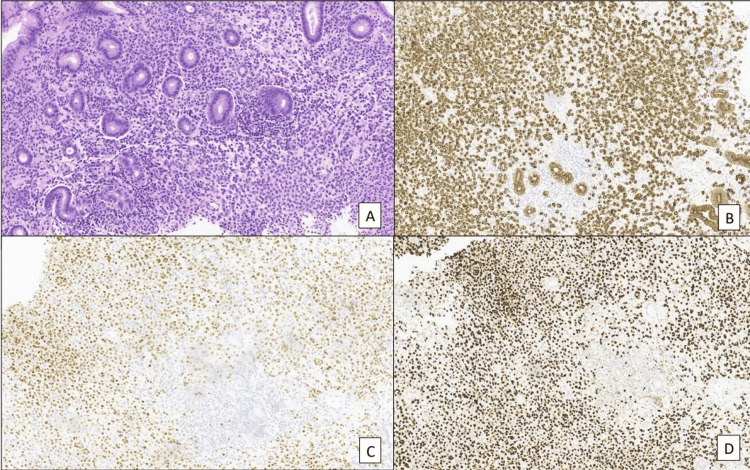
Gastric biopsies A) haematoxylin-eosin; B) cytokeratin Ae1ae3; C) estrogen receptor; D) GATA 3

Gastric biopsies revealed diffused infiltration of the gastric mucosa from poorly cohesive adenocarcinoma with signet ring cells (Figure 4). Immunochemistry was positive for PanCK, GCDF15, GATA3, ER, and PgR and negative for TTF1, CK20, CA19-9, CA125, vimentin, and E-cadherin.; HER2 overexpression was equivocal, with the fluorescence in situ hybridisation (FISH) test being negative; Ki-67 was 10%.

The microscopic findings combined with the ER, PgR, and GATA3 positivity and CK20 negativity were consistent with a diagnosis of metastatic lobular breast cancer.

**Figure 5:**
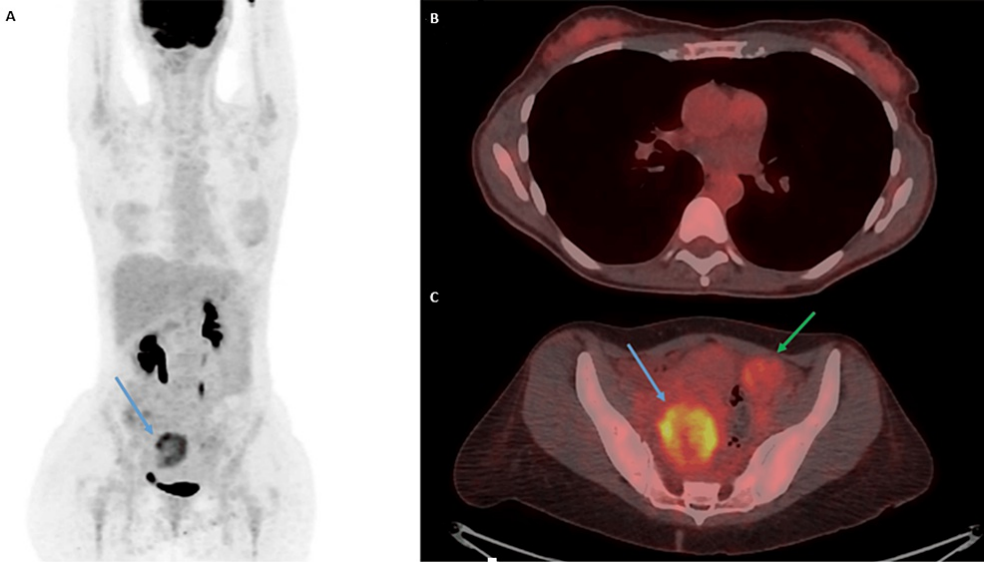
Positron emission tomography/computed tomography The maximum intensity projection image shows only a significant uptake in the right pelvic region (panel A, blue arrow). Axial positron emission tomography/computed tomography images show a mild diffuse uptake in the bilateral mammary gland without observing clear pathological foci, probably physiological (panel B). Axial positron emission tomography/computed tomography image in which a moderated uptake is identified in the right ovarian lesion (blue arrow), observing a lesser uptake in the left ovarian lesion (green arrow), similar to the surrounding background activity (panel C).

F-fluorodeoxyglucose (FDG)-positron-emission tomography (PET) demonstrated intermediate FDG uptake of the right ovarian mass and low uptake of the left ovarian mass. In addition, the PET scan revealed low uptake in the ascending colon and the gastric wall that was similar to the background activity. There was also mild breast diffuse uptake without any discrete lesions (Figure 5).

**Figure 6:**
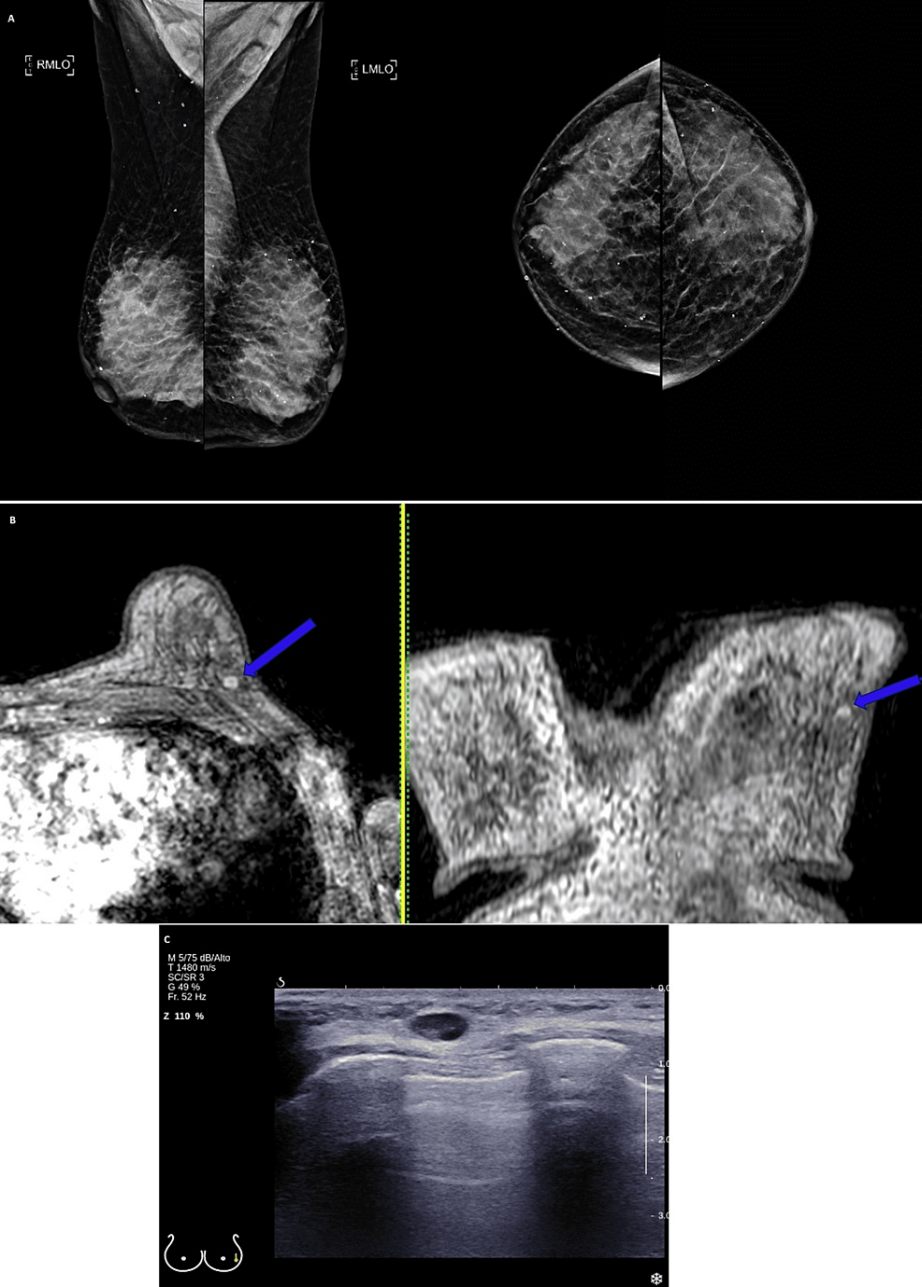
Mammogram, breast magnetic resonance imaging, and breast ultrasound On mammograms, dense breasts are observed (American College of Radiology Breast Density Category D), with no evidence of dominant nodules, suspicious microcalcifications, or distortions in the glandular architecture (panel A). On magnetic resonance imaging, the morphological analysis reveals an extension of glandular tissue towards the left axillary region, consistent with an ectopic gland. A nodular enhancing area of 4 x 5 x 4 millimetres is identified in the axillary extension of the left breast, classified as Breast Imaging Reporting & Data System category (BI-RADS) 3. It is difficult to characterize due to the marked background glandular enhancement, which is likely to correspond to an intramammary lymph node (panel B, blue arrows). On breast ultrasound, a reactive-looking lymph node is observed in the left axillary extension and another one in the upper outer quadrant of the left breast. No other significant findings are seen in the rest of the ultrasound examination of the left breast (panel C).

A breast work-up was then performed. A mammogram and breast ultrasound revealed no nodules, microcalcifications, asymmetry, or distortions. Axillary lymph nodes were normal-sized (Figures 6A, 6C). A breast MRI showed a 4 mm nodule on the left breast that was compatible with the intramammary lymph node without abnormal characteristics (Figure 6B).

Although the clinical presentation and the radiological findings suggested metastatic cancer of gastrointestinal origin, the histopathological findings were consistent with metastatic lobular breast cancer. The patient was referred to medical oncology for further treatment. At the time of the referral, the patient had abdominal pain, constipation, anorexia, significant ongoing weight loss, and ascites. First-line hormone treatment with goserelin every four weeks, letrozole, and palbociclib was started. After one month of treatment, the patient’s symptoms significantly improved; constipation had resolved, and abdominal pain and anorexia had improved. The patient’s ascites was significantly reduced, and her body weight was stabilised. After three months of treatment, CT scans showed evidence of a partial response, as the ascites had resolved and the sizes of pelvic masses had reduced.

Twenty-one months after the initial presentation, the patient remains on treatment with goserelin, letrozole, and palbociclib without any evidence of clinical or radiological progression. 

## Discussion

This case highlights the obstacles encountered by clinicians when diagnosing lobular breast cancer. The atypical clinical presentation with no palpable breast nodule and sometimes the absent radiological breast and axillary radiological findings combined with the distinct metastatic pattern can make the diagnosis challenging. In this case, the patient presented with non-specific constitutional symptoms of progressive weight loss, anorexia, and fatigue, symptoms consistent with abdominal pathology. No palpable breast nodule was reported, and the latest screening mammogram was normal. On clinical examination, there was a palpable abdominal mass, while the breast examination was unremarkable. The history of the presenting complaint and the radiologic and endoscopic findings were consistent with possible metastatic cancer of gastrointestinal origin with no evidence of breast abnormalities. The biopsy results, however, indicated that the metastatic sites were of breast origin, with histopathologic findings being typical of metastatic lobular breast cancer with the presence of ER and PgR. Despite the exhaustive mammographic workup, including mammogram, ultrasound, and MRI, no primary breast lesion or axillary lymphadenopathy was detected.

The unique growth pattern of ILC makes its diagnosis via mammogram difficult. Invasive lobular breast cancer infiltrates the breast tissue without significantly changing the normal breast tissue with a lack of desmoplastic response; thus, it is more difficult to detect ILC than other histological subtypes. The most common mammographic findings of ILC are spiculated masses, architectural distortions, asymmetry, and calcifications [3].

The overall sensitivity of MRI for the detection of ILC ranges from 83% to 100%, but at the cost of low specificity. The radiological characteristics of ILC vary. The most common radiological findings are an irregular mass with type 2 enhancement, followed by non-mass lesions with different patterns. The increased sensitivity of MRI has improved the detection rate of ILC and the diagnosis of synchronous and multifocal lesions, which improves surgical planning [14].

The cardinal histologic finding of ILC is dispersed strands of tumour cells organised in single files due to the loss of E-cadherin, which is an important component of cell-cell adhesion. E-cadherin is essential for the normal functioning of breast and gastric tissue, as it regulates tissue homeostasis. Although E-cadherin is a useful marker, its presence should not preclude the diagnosis of ILC. Typically, ILC overexpresses ER and PgR with negative HER2 overexpression [15].

The staging recommendations for other breast cancer histologic subtypes also apply to ILC. However, gastrointestinal symptoms or atypical central nervous system (CNS) symptoms should alert the treating physician to the presence of metastatic disease due to the atypical spread pattern. Invasive lobular breast cancer has the tendency to metastasize to the gastrointestinal tract, peritoneum, ovaries, and leptomeninges [16]. The use of FDG-PET has shown promising results for the detection of distant diseases, although it is not routinely recommended. Oestrogen receptor-targeting PET tracers may further improve the detection rates of metastatic lesions [17]. In our case, only the metastatic ovarian implants were FDG-avid. 

The systemic treatment of ILC is similar to the treatment of other histological subtypes. Invasive lobular breast cancer is notoriously chemoresistant, and hormone treatment in combination with CDK4/6 inhibitors yields a better response and improves survival [18], though patients do eventually receive chemotherapy. 

The management of breast cancer patients with peritoneal carcinomatosis remains challenging. The presence of peritoneal carcinomatosis is a poor prognostic factor among patients with metastatic breast cancer and a significant cause of morbidity with symptoms such as ascites, bloating, or intestinal obstruction. Currently, there is limited data available on the efficacy of local treatments such as hyperthermic intraperitoneal chemotherapy (HIPEC) or surgical intervention. As a result, treatment strategies primarily rely on systemic options, including hormone therapy and chemotherapy [19].

In this case, the patient responded clinically and radiologically to first-line hormone treatment in combination with CDK4/6 inhibitors. Although the patient was symptomatic with significant weight loss, abdominal pain, and partial colon obstruction, first-line hormone treatment was preferred instead of chemotherapy due to the low response rate of ILC to cytotoxic treatment and the good efficacy of CDK4/6 inhibitors in patients with impending or established visceral crises. Recent data suggest that first-line treatment with CDK4/6 inhibitors improves overall survival in patients with an impending or established visceral crisis [20].

## Conclusions

This case demonstrates the challenges in the diagnosis of ILC due to its unique spread pattern and atypical presentation. Although rare, metastatic lobular breast cancer without an identifiable primary, despite exhausting breast imaging work-up, is a known clinical entity. In this case, the absence of axillary lymph node involvement made the diagnosis challenging. The diagnosis was mainly guided by the histologic findings combined with the metastatic pattern of peritoneal carcinomatosis. While symptomatic and despite the significant metastatic tumour burden, the patient responded to first-line hormone treatment combined with CDK4/6 inhibitors. Future studies should improve the understanding of the molecular mechanisms that lead to the development of ILC and should include trials focused on this subgroup of patients.
